# Physical Exercise as a Therapeutic Approach in Gastrointestinal Diseases

**DOI:** 10.3390/jcm14051708

**Published:** 2025-03-03

**Authors:** Juliana Soares Severo, Alda Cássia Alves da Silva, Brenda Lois Barros dos Santos, Thiago Sousa Reinaldo, Aureliano Machado de Oliveira, Rodrigo Soares Pereira Lima, Francisco Leonardo Torres-Leal, Armênio Aguiar dos Santos, Moisés Tolentino Bento da Silva

**Affiliations:** 1 Graduate Program in Food Sciences and Nutrition, Metabolic Diseases, Exercise and Nutrition Research Group (DOMEN), Laboratory of Metabolic Diseases Glauto Tuquarre, Department of Biophysics and Physiology, Center for Health Sciences, Federal University of Piaui, Teresina 64049-550, PI, Brazil; julianasevero@ufpi.edu.br (J.S.S.); aureliano@frn.uespi.br (A.M.d.O.); rodrigo.soaares@ufpi.edu.br (R.S.P.L.); torresleal@ufpi.edu.br (F.L.T.-L.); 2 Graduate Program in Pharmacology, Federal University of Piauí, Teresina 64049-550, PI, Brazil; aldacassiia.ads@ufpi.edu.br; 3 Graduate Program in Pharmaceutical Sciences, Federal University of Piauí, Teresina 64049-550, PI, Brazil; brendaloissantos@gmail.com; 4 Multicenter Postgraduate Program in Physiological Sciences in Association with the Brazilian Society of Physiology, Federal University of Piauí, Teresina 64049-550, PI, Brazil; thiago.reinaldo@ufpi.edu.br; 5 Department of Physiology and Pharmacology, Federal University of Ceará, Fortaleza 60430-270, CE, Brazil; meno@ufc.br; 6 Laboratory of Physiology, (MedInUP/RISE-Health)—Department of Immunophysiology and Pharmacology, School of Medicine and Biomedical Sciences, University of Porto, 4050-313 Porto, Portugal

**Keywords:** exercise, gastrointestinal diseases, therapeutics

## Abstract

**Background/Objectives**: Physical exercise can have significant consequences for the gastrointestinal tract, which is why there have been studies into its influence on the treatment of conditions such as colorectal cancer, inflammatory bowel diseases (IBD), and irritable bowel syndrome (IBS), being that there is epidemiological evidence that exercise has a protective effect against colon cancer. This review aims to demonstrate the mechanisms of action of physical exercise in the gastrointestinal tract, as well as the benefits of exercise in diseases associated with the digestive system, in addition to gathering training recommendations in treating different gastrointestinal diseases. **Results:** Physical exercise modulates gastrointestinal motility, permeability, immune responses, and microbiota composition, with both beneficial and adverse effects depending on intensity and duration. Regular moderate exercise is associated with improved quality of life in IBD and IBS, reduced colorectal cancer risk, and potential symptom relief in constipation. However, high-intensity exercise may exacerbate gastroesophageal reflux symptoms and increase the risk of gastrointestinal bleeding. While aerobic exercise has been extensively studied, the effects of resistance training on gastrointestinal health remain underexplored. **Conclusions**: New methodologies and techniques, such as molecular biology and the study of gastric receptors, have led to advances in understanding the gastrointestinal changes associated with physical exercise. These advances cover different exercise intensities and are being investigated in both experimental models and clinical studies.

## 1. Introduction

Research involving exercise physiology has long focused on the responses and adaptations of the respiratory, cardiovascular, neuroendocrine, and neuromuscular systems, considering that many health and quality-of-life benefits of regular exercise arise from these systems’ responses and adjustments [1,2]. Another area of study that deserves significant attention concerns the systemic repercussions of physical exercise, particularly its effects on the gastrointestinal tract [3,4,5].

After the first experiments performed by Beaumont on gastrointestinal function during exercise during the 1800s, which were never published, Anton Julius Carlson, an American Physiologist, became a pioneer in publishing on the subject [6]. After Carlson, research on exercise and the gastrointestinal tract was sparse. However, in the late 1960s, driven by the consumption of energy drinks and the practice of running, there was a greater interest in the study of gastrointestinal function and physical exercise. From the 1980s onwards, several researchers began to investigate the effects of exercise on the gastrointestinal tract through clinical and epidemiological studies, particularly in terms of harmful effects [7].

Among these repercussions, gastrointestinal symptoms in physical exercise practitioners and athletes are typical, such as nausea, diarrhea, vomiting and intestinal bleeding, particularly in high-intensity training. On the other hand, physical exercise also prevents several diseases related to the gastrointestinal tract, such as colon carcinoma, diverticulitis, cholelithiasis, and constipation. Thus, several studies have demonstrated an interest in the relationship between exercise and the gastrointestinal tract, with an emphasis on the prevention and treatment of diseases, as well as the optimization of athletic performance, given that the stomach and intestines are essential for the digestion and absorption of macronutrients and micronutrients required by active muscles [8,9].

Regarding the mechanisms related to the effects of exercise on the gastrointestinal tract, its impact on the composition of the intestinal microbiota, its antioxidant and anti-inflammatory action, which may have protective action, reducing permeability in the gastrointestinal tract [10,11,12]. In this sense, this review aimed to (1) demonstrate the mechanisms by which physical exercise acts on the gastrointestinal system; (2) show recent evidence of the effects of training in the treatment of diseases of the gastrointestinal tract, such as gastroesophageal reflux disease (GERD), irritable bowel syndrome (IBS), inflammatory bowel diseases (IBD), and colorectal cancer; and (3) about training recommendations in the treatment of different gastrointestinal diseases.

## 2. Results

### 2.1. Basic Concepts About Physical Exercise

According to the World Health Organization [13], physical inactivity is the fourth leading risk factor for global mortality, accounting for 5.5% of deaths per year, among various risk factors such as dyslipidemia, obesity, and hypertension. Research highlighting the importance of an active lifestyle is growing exponentially and supports the notion that improved quality of life is associated with reduced periods of physical inactivity [14].

Physical activity recommendations vary based on intensity and duration. According to various health institutions, it is recommended to engage in 150 min of moderate physical activity per week, 75 min of vigorous activity, or a combination of these intensities [15]. These levels are sufficient to provide health benefits. However, when weekly exercise time is increased to 300 min, there is an additional advantage in reducing overall mortality, suggesting that a higher volume of physical activity may have even more significant benefits [16]. Another way to monitor physical activity levels is through daily step counting. Studies show that walking more than 7000 steps per day significantly reduces the risk of various conditions related to physical inactivity, such as cardiovascular diseases, diabetes, and obesity [17,18].

The literature consistently shows that regular physical exercise is associated with a reduced risk of premature mortality and contributes to the prevention of more than 25 pathologies, including chronic medical conditions such as hypertension, breast and colon cancer, type II diabetes, gestational diabetes, and gallstones. A dose-dependent correlation between physical exercise and primary and secondary prevention of chronic disorders has been demonstrated, in addition to a positive association with adolescent health [19,20].

In this way, the WHO introduced physical activity and physical exercise in the World Public Health Agenda, launching the “General Strategy for Food, Physical Exercise and Health” and describing actions necessary to increase physical activity globally [21]. In addition to the “Global Physical Activity Recommendations for Health”, published in 2010, highlighting the primary prevention of Chronic Noncommunicable Diseases (CNDs) through physical activity [22].

In this sense, the WHO defines physical activity as any body movement produced by skeletal muscle that results in energy expenditure above the rest, including physical activity practice during work, playing, and domestic activities. Physical exercise is any planned activity, structured and systematized, involving body movements of skeletal muscle of contraction and relaxation. It aims to improve physical fitness components, using energy substrates above resting values [23].

Physical exercise can be characterized in two ways: (i) intermittent and (ii) continuous. Regarding intermittent exercise, its primary energy source is the phosphagen system, which is the fundamental fuel for short-duration, high-intensity activities such as 100 m sprints in athletics, 50 m and 100 m swimming, weightlifting, and cycling sprints, among others [24]. The ATP-Creatine Phosphate (CP) energy system provides a rapid but short-lived energy source, depleting its stores in approximately 10 s. During high-intensity activity, ATP is broken down into ADP and inorganic phosphate (Pi) by ATPase, releasing energy. Creatine phosphate (CP) serves as an energy reservoir, donating its phosphate group to ADP via creatine kinase (CK) to rapidly regenerate ATP, ensuring continued energy supply for short-duration, high-power activities [25].

In the phosphagen system, intense exercise can lead to the development of acidosis, increasing the reduction of pyruvate to lactate and reducing the proton transport capacity via NADH+ (nicotinamide adenine dinucleotide), thus increasing lactate production and the release of free hydrogen ions (H+). Suppose the buffering capacity of this proton is overwhelmed. In that case, it results in a decrease in pH, which can cause fatigue through several mechanisms, such as plate acidosis, the inhibition of phosphofructokinase, the inhibition of the SERCa (Sarcoplasmic Endoplasmic Reticulum Ca^2^⁺-ATPase) pump, lower calcium conductance, reduced troponin/tropomyosin interaction, and the stimulation of type C fibers in the central nervous system, inducing a “burning” discomfort [26,27].

The aerobic system uses the oxidative phosphorylation system as an energy source for ATP production with subsequent energy generation. This energy system is utilized in long-duration, low-intensity exercises requiring greater aerobic capacity, such as 5000 m and 10,000 m races, marathons, open-water swims, and long-duration cycling events [28].

Exercise results in numerous changes in the gastrointestinal tract (metabolic improvements, reduction in chronic systemic inflammation, lower serum insulin levels, improvements in the gut microbiota associated with preservation of the intestinal barrier and improved bile acid homeostasis). Most of these effects depend on the volume and intensity applied, although low-intensity exercises do not describe significant damage [29]. Extreme exercises and dehydration states are reported as causes of gastrointestinal symptoms by 70% of athletes, and intestinal ischemia is considered the leading cause of nausea, vomiting, abdominal pain, and diarrhea [30].

### 2.2. Gastrointestinal Motility and Exercise

Gastrointestinal motility results from the activity of the musculature of the digestive tract. This intrinsic muscle activity is called motility. This varies according to the segment and circumstances, notably the dietary condition. Segmentation contractions predominate in motor behavior shortly after food ingestion, where neighboring gastrointestinal segments contract simultaneously and continuously [31]. Already under fasting, the migratory motor complex occurs, a pattern of cyclic and consecutive motility, passing from the stomach until reaching, about 90 min later, the ileocecal valve. Given the similarity in morphology, such variation in activity stems from neurohumoral regulatory mechanisms [32,33].

The motility of the gastrointestinal tract is primarily coordinated by the neurons of the myenteric plexus, which are present along the gastrointestinal tract. The myenteric plexus regulates peristalsis by modulating muscle wall contraction, both the frequency and intensity of contraction. The essential stimulus for the myenteric plexus comes from the mechanical distension of the gastric wall through food, as well as from the irritation of the epithelium and the activity of the extrinsic nervous system [34]. Although the human stomach is anatomically a single viscera, its motor behavior is quite distinct in the proximal and distal portions. In the proximal portion, the accommodation of the ingested food occurs without significant changes in intraluminal pressure, thanks to receptive relaxation. After the gastric secretions are mixed, the food passes to the distal stomach, from where it is emptied and gushes into the small intestine [35].

With its slow, sustained contractions, the proximal stomach plays a key role in regulating intragastric pressure and gastric emptying of liquids. In contrast, the distal stomach, with its peristaltic contractions, plays a crucial role in mixing gastric secretions with food and in the grinding process, especially in the gastric emptying of solids [36].

Through vigorous contractions that even occlude the stomach lumen, the chyme advances towards the pylorus, which, when contracted, prevents the passage of solids, which undergo retropulsion and are progressively crushed until liquefaction. Once in liquid form, the material is quickly evacuated in the interval between waves of contractions, thanks to gastric tone. Therefore, the sensory perception of gastric fullness is correct, depending on the nature of the food, as liquids are emptied more quickly and solids more slowly. On the other hand, the indigestible material is only emptied from the stomach when the migrating motor complex takes control during fasting [37,38].

Physical exercise per se promotes physiological adjustments, whether in the neuromuscular, cardiovascular, respiratory, endocrine systems or even in the gastrointestinal tract. However, such adjustments do not occur uniquely and linearly in the various systems, as they depend on the exercise’s time, intensity, volume, nature, and energy sources governing such activities [39].

Another critical point of exercise on the gastrointestinal tract is related to the possible risks and benefits caused by this practice. In this sense, the impact of exercise and physical activity on the gastrointestinal tract has taken the scientific community’s interest in an emerging way. For more than two decades, research has focused primarily on the risks of strenuous exercise, especially gastrointestinal symptoms. However, over the past few years, interest has also turned to the potential benefits of physical exercise on the gastrointestinal tract. Several studies indicate an inverse relationship between physical exercise and the risk of gastrointestinal diseases, such as colon cancer, diverticulitis, colitis, and constipation [40,41,42,43]. Table 1 shows some risks and benefits of exercise in gastrointestinal tract. 

Acute physical exercise influences gastric motility in a dose-response relationship with intensity, with low-intensity exercises seeming to accelerate the gastric emptying rate. In contrast, there is a delay in gastric emptying at high intensity. Different modalities, such as the volume of food intake, the osmolality of energy drinks, and the duration of exercise, also seem to affect gastric motility [44].

Studies indicate the direct benefits of aerobic exercise on the gastrointestinal tract [45,46,47]. Resende et al. [45] highlight that moderate-intensity exercise can improve VO_2_ peak and positively influence gut microbiota composition in non-obese men. Specifically, the study observed an increase in the relative abundance of Streptococcus and a decrease in an unclassified genus from the *Clostridiales* order. Additionally, VO_2_ peak was positively associated with *Roseburia*, *Sutterella*, and *Odoribacter*, while BMI negatively correlated with *Desulfovibrio* and *Faecalibacterium*. These microbial changes suggest potential benefits for gut function, including enhanced short-chain fatty acid (SCFA) production, improved gut barrier integrity, and reduced inflammation, all of which may contribute to better digestion and metabolic regulation. Performing moderate-intensity aerobic exercise on a treadmill (at 75% of maximum heart rate) for 25 min increased gastric compliance in healthy men and women, which refers to the stomach’s ability to expand in response to food or liquid intake while maintaining intragastric pressure. Still, it did not alter these individuals’ satiety perception [48]. Changes in gastric compliance can be attributed to neuroendocrine adaptations promoted by exercise [48,49]. It is noteworthy that physical exercise can induce repercussions on the autonomic nervous system, thus influencing the gastrointestinal tract. The vagus nerve mediates an increase in function via the parasympathetic nervous system, which reduces sympathetic excitability and maintains sympathovagal balance [50,51].

In another study, Carvalho et al. [52] found that acute anaerobic exercise was able to increase gastric accommodation, which refers to the stomach’s ability to relax and expand in response to food intake and reduce satiety in healthy men, these effects being mediated by the secretion of lactate, CK and some plasma cytokines, such as interleukins (IL)-6, -13 and tumour necrosis factor α (TNF-α). In addition, one of the possible explanations for this phenomenon concerns the activation of cholinergic pathways that increase gastric tone and the release of nitric oxide, promoting increased gastric accommodation mediated by physical exercise [52,53]. Table 2 shows common perceptions of the gastrointestinal effects of exercise and their scientific evidence.

### 2.3. Intestinal Permeability and Exercise

Practitioners of physical exercise, especially those of long duration, such as marathons, triathlons, and adventure races, present significant biochemical and physiological changes, which have become the target of research for a better understanding of these phenomena aiming at better performance. The manifestation of gastrointestinal symptoms in athletes is one of the most common causes of loss of sports performance in training and competitions. Some studies have pointed to a range of 30 to 83% of gastrointestinal symptoms among runners, mainly considering complaints related to the lower gastrointestinal tract (diarrhea, rectal incontinence, rectal bleeding, and abdominal pain). In addition, women are more susceptible to these problems when compared to men, and some modalities report more complaints, such as cyclists and triathletes [11,68].

The type of exercise and training variables related to intensity and volume of food intake during exercise play a fundamental role in the etiology of gastrointestinal injuries [69]. Although exercise dramatically influences the entire gastrointestinal tract, the intestinal segments deserve greater attention, considering that through them, nutrients are absorbed, and most of the gastrointestinal symptoms associated with exercise alter the intestinal permeability [70]. Changes in intestinal permeability refer to the diffusion-mediated passage of molecules larger than 150 Da through the intestinal barrier, particularly via tight junctions and other intercellular pathways, as desmosomes [71].

Tight junction proteins are key regulators of paracellular transport in the intestinal barrier, including claudins, occludins, and zonulins. The dysregulation of these proteins can lead to increased intestinal permeability, compromising barrier integrity. One of the primary factors contributing to this dysfunction is alterations in the gut microbiota, which influences tight junction expression, intestinal immune responses, and exercise-induced thermal stress [3,68,70].

There are many causes of changes in intestinal permeability caused by exercise, such as mechanical factors, where it is well described that runners suffer from intestinal disorders caused by mechanical forms of acceleration/deceleration, as well as neuromuscular alterations resulting from psoas muscle hypertrophy, which presses the gastrointestinal tract, generating gastrointestinal symptoms [69].

The mesenteric circulation and gastrointestinal symptoms in long-distance runners may be influenced by mesenteric lymphatic vessel contractility, and probiotics present significant diagnostic and therapeutic challenges [72]. Smarkusz-Zarzecka et al. [72] conducted a randomized controlled trial evaluating the effects of a multi-strain probiotic supplement on gastrointestinal symptoms and serum biochemical parameters in long-distance runners. After a 3-month intervention, participants in the probiotic group reported a reduction in constipation, with women experiencing greater overall health improvements than men. However, no significant changes were observed for diarrhea, reflux, or IBS-like symptoms compared to the placebo. These findings suggest that probiotics may play a role in modulating gut function and systemic metabolism, particularly in endurance athletes prone to gastrointestinal disturbances.

Many athletes use aspirin or other non-steroidal anti-inflammatory drugs (NSAIDs) for analgesia. While effectively controlling pain, NSAIDs disrupt oxidative phosphorylation and inhibit cyclooxygenase (COX) enzymes in the gastrointestinal mucosa. In addition to these effects, NSAIDs likely contribute to cytoskeletal disruption and impaired calcium homeostasis, generating free radicals that cause oxidative damage. This process may weaken tight junctions and desmosomes, further compromising intestinal barrier integrity. Prolonged or intense exercise, when combined with NSAID use, exacerbates intestinal permeability, as demonstrated by Van Wijck et al. [73].

Prolonged exercise especially for endurance runners, can increase gastrointestinal symptoms’ incidence and severity [44]. The occlusion of blood flow can be improved with a higher intensity and/or duration of exercise and/or environmental heat stress [74]. The transient ischemic state during physical exercise causes damage to the GI tissues, triggering an inflammatory response. Studies show that when blood flow is restored—reperfusion—cells continue to undergo necrosis, and a cascade of inflammatory mediators (pro-inflammatory cytokines, neutrophils, adhesion molecules) are signaled to repair the damaged tissue [75].

**Figure 1:**
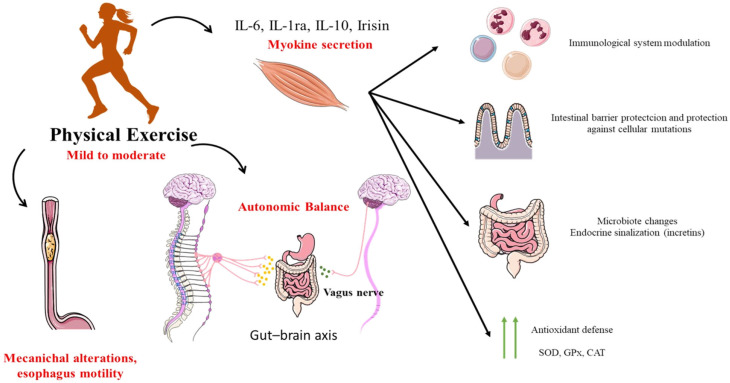
Regular physical exercise modulates the beneficial effects in different systems, preventing and auxiliary such as therapy form in gastrointestinal disorders. Physical exercise promotes the release of myokines, which can act in the modulation of the immune system, in the maintenance of the intestinal epithelial barrier and protection against mutations, and by promoting changes in the microbiota and endocrine signaling and increasing the antioxidant defense. Another physical exercise action modulates the autonomic balance, regulating the sympathetic and parasympathetic functions that innervate the gastrointestinal tract. In addition, the mechanical changes promoted by physical exercise can alter gastroesophageal motility in a dose–response relationship with intensity, and low-intensity exercises seem to induce an acceleration of the gastric emptying rate. In contrast, there is a delay in gastric emptying at high intensity.

Another important factor related to permeability is intestinal ischemia. This can occur as early as 10 min after performing high-intensity exercise, measured by gastric tonometry. Splanchnic hypoperfusion for 20 to 60 min in a cyclist at 70% VO_2_max intensity, followed by 10 min of reperfusion, causes rapid ATP breakdown to AMP, activating hypoxanthine. During the reperfusion cycle, hypoxanthine is reduced to xanthine by the calcium-activated enzyme xanthine oxidase. The increase in calcium may result from calcium pump dysfunction during ischemia. Xanthine oxidase releases hydrogen peroxide, a potent free radical that causes tissue disruption and the breakdown of tight junction proteins. Thermal stress during exercise can also lead to the impairment of tight junction proteins, which are responsible for maintaining the morphological structure of intestinal cells and the gastrointestinal barrier function [76,77]. These effects of physical exercise on the gastrointestinal tract can be seen in Figure 1.

### 2.4. Gastrointestinal Disease and Exercise

On the other hand, exercise can be beneficial in treating several gastrointestinal disorders. Table 3 summarizes studies exploring physical activity’s effects on various gastrointestinal conditions.

#### 2.4.1. Gastroesophageal Reflux Disease

Gastroesophageal reflux disease (GERD) is a gastrointestinal motility dysfunction characterized by the irregular reflux of stomach contents leading up to the esophagus, causing mucosal damage and several other symptoms [84]. Heartburn (pyrosis), a burning sensation or discomfort due to increased acidic juice in the stomach with pain and bitter taste in the mouth, and regurgitation, characterized by the involuntary return of gastric contents to the esophagus, are common in GERD, accompanied by extraesophageal symptoms such as teeth erosion, epigastric pain, laryngitis, and cough [85,86]. GERD affects the quality of life of individuals and can progress to erosive esophagitis, esophageal stricture, Barrett’s esophagus, and esophageal adenocarcinoma [87]. Patient improvement is associated with the use of proton pump inhibitor drugs, the first line of drugs used for treatment, and the modification of the patient’s lifestyle [88].

Light to moderate physical exercise generates benefits for GERD, while high-intensity exercise, depending on the type and duration, can be considered a risk to the patient’s health [56,89]. A fibre-rich and low-fat-rich diet is also recommended to prevent reflux symptoms [90]. The inspiratory of diaphragmatic muscle training helps improve the pressure on the gastroesophageal junction, reducing the progression and symptoms of the disease [91]. It is considered that the lower esophageal sphincter region is responsible for the prevention of gastroesophageal reflux, and because of this, respiratory training exercises on the diaphragm indicate improvement in patients evaluated by esophageal pH monitoring, quality-of-life scores and use of proton pump inhibitors [92,93,94].

High-intensity physical activity increases the possibility of reflux episodes in patients affected by the erosive form with a positive correlation of VO2 ≥ 70% in a stress exercise test; however, light and short-term exercises do not affect the occurrence of reflux, even in patients with overweight or obesity [83]. Exercise causes worsening symptoms of gastroesophageal reflux related to a decrease in blood flow by about 80% by activating adrenergic receptors in athletes and untrained individuals. It may intensify symptoms during dehydration [9,95]. Usually, some athletes have oesophagal reflux in high-intensity exercises, which require a lot of physical effort and prolonged duration, associated with a worsening in postprandial exercises, which increases with the increase in resistance. GERD presents complexity in its symptomatology to other upper gastrointestinal diseases, making it difficult to differentiate from angina, and may also increase asthma symptoms [96].

Collings et al. [79] analyzed the effect of running, cycling, and weightlifting on 10 subjects from each sport with a three-month clinical history of heartburn during exercise. Exercises were standardized at 65% (60 min) and 85% (20 min) of maximum heart rate, and effects were observed in fasting or after a 15-min interval diet. Worsening of gastroesophageal reflux was observed in postprandial cases compared to fasting. Within the three modalities, weightlifting showed more significant reflux and heartburn. Running showed symptoms and moderate reflux, while cycling showed little worsening of the condition. It is reiterated that intense physical exercise causes considerable reflux and worsens other symptoms in athletes.

In this context, running, rowing, weightlifting, and cycling athletes have common upper gastrointestinal characteristics such as heartburn, epigastric pain, regurgitation, nausea, and vomiting. GERD is the primary disease that causes these symptoms in the upper gastrointestinal tract in athletes. The symptomatology of the disease is usually caused by exercise; GERD by exertion affects more individuals who have GERD at rest. However, it can affect only athletes when associated with physical exercise [97,98].

Corroborating the study, ten healthy volunteers who practiced some physical activities were submitted to a treadmill for 30 min (60% of maximum heart rate) with rest and then running for 20 min (85% of maximum heart rate) before exercise. A standardized meal. The pH of the esophageal region was much more acidic (<4), and there was a decrease in esophageal sphincter pressure, a reduction in peristaltic contractions causing more significant episodes of reflux [54].

In this context, athletes in sports such as running, rowing, weightlifting, and cycling commonly exhibit gastrointestinal characteristics such as heartburn, epigastric pain, regurgitation, nausea, and vomiting. GERD is identified as the primary condition causing these symptoms in the upper gastrointestinal tract in athletes. The symptoms of the disease are usually triggered by exercise, and exercise-induced GERD affects individuals who already have GERD at rest; however, it can affect athletes only when associated with physical exercise [99,100].

#### 2.4.2. Inflammatory Bowel Disease (IBD)

Inflammatory bowel diseases (IBD) comprise two primary forms of clinical manifestation: Crohn’s disease, which is characterized by transmural inflammation of the intestinal mucosa, consisting of any part of the gastrointestinal tract from the mouth to the anus, and ulcerative colitis, which involves only inflammation in the colonic mucosa, with more significant activity in the rectal region [101,102].

The chronic inflammation characteristic of IBD involves the mucosal and submucosal layers of the gastrointestinal tract, leading to symptoms such as bleeding, abdominal pain, diarrhea, malnutrition, and increased intestinal permeability [101]. Among the factors involved in the pathogenesis of these diseases, genetics, dysregulation in the intestinal microbiota, and environmental factors such as diet and physical inactivity stand out [103,104].

Physical exercise improves the inflammatory condition of Crohn’s disease and ulcerative colitis. The regular practice of moderate-intensity walking or running three times a week for 10 weeks in patients with Crohn’s disease with mild to moderate disease activity appears to improve these individuals’ quality of life and well-being [105]. Walking three times a week at 60% of maximum heart rate enhanced patients’ quality of life with Crohn’s disease without exacerbating disease activity [40]. Additionally, physical exercise provides further benefits, including improving gut microbiota composition and exerting antioxidant and anti-inflammatory effects, which may have a protective role in reducing gastrointestinal permeability [8,10,41].

Data on the relationship between physical exercise and IBD are more consistent for Crohn’s disease compared to the scarce research developed with patients with ulcerative colitis. In a study developed in the United Kingdom, Chan et al. [106] observed that patients with Crohn’s disease and ulcerative colitis have limitations in physical exercise. However, patients who regularly practice some sports report improvements in the disease symptoms.

The performance of physical exercise leads to increased physical fitness, bone mineral density, quality of life, and reduced stress and anxiety associated with IBD without adverse effects related to exercise interventions [107]. It is essential to mention that training recommendations for patients with IBD should be lower than those for healthy individuals due to the inflammatory responses induced by exercise, particularly high-intensity exercise [10]. Performing acute low-intensity exercise or high-intensity interval exercise in pediatric patients with Crohn’s disease also does not appear to exacerbate the inflammatory condition of the disease [108].

According to the European Crohn’s and Colitis Organization, Pérez [109] recommends exercise for Crohn’s disease. As for aerobic activity, 20–30 min of low-intensity walking is recommended, at 60% of maximum heart rate, 3 days a week and evolving according to the patient’s progress. Regarding resistance training, using elastic bands or free weights is recommended, as it is an individualized program according to age, conditioning level, goals, and exercise preferences. Elsenbruch et al. [110] found improvement in quality of life in patients with ulcerative colitis in remission, with no changes in clinical and physiological parameters after a structured program of moderate-intensity physical exercise, Mediterranean diet, and cognitive behavioral therapy once a week for 10 weeks.

Physical exercise improves the quality of life. It can be considered an alternative therapy in IBD due to the role of skeletal muscle in the secretion of myokines such as irisin, IL-6, IL-15, and IL-1ra released during and after the exercise session. Regarding irisin, it is vital in regulating metabolic functions in adipose tissue, which are altered in the presence of Crohn’s disease and ulcerative colitis [10]. Myokines play a crucial role in modulating gut microbiota composition and gastrointestinal function. Irisin, a myokine released during exercise, has been shown to influence gut microbiota, exerting anti-inflammatory effects within the gastrointestinal tract. Additionally, muscle activity enhances the synthesis of brain-derived neurotrophic factor (BDNF), which may help protect intestinal cells against mutations, promoting gut health and overall homeostasis. Furthermore, certain interleukins (ILs) function as anti-inflammatory mediators and play a role in appetite regulation within the gastrointestinal tract. This endocrine signaling network, which links skeletal muscle and gut function, is called the muscle–gut axis [10,52].

Another substance secreted by skeletal muscle that deserves to be highlighted is IL-6 since, despite being considered a pro-inflammatory factor, it induces anti-inflammatory responses such as an increase in IL-10 and IL-1ra and stimulates the secretion of peptides like glucagon 1 (GLP-1), which may act in the repair of the intestinal mucosa after damage related to the pathogenesis of IBD [10].

In addition, exercise seems to play an essential role in improving the diversity of the intestinal microbiota, which is usually dysregulated in Crohn’s disease and ulcerative colitis, being one of the factors that can aggravate or predispose to the emergence of these diseases [3,10,111]. In a systematic review, Koutouratsas et al. [112] show pre-clinical and clinical studies that regular exercise increases the presence of beneficial bacteria, such as Bacteroidetes, Clostridium septum, *Prevotella*, *Bifidobacterium*, and *Roseburia*, while reducing the abundance of *Proteobacteria*, a group often linked to intestinal inflammation. These changes promote the production of SCFAs, strengthen the intestinal barrier, modulate the immune response, and inhibit pathogenic bacterial adhesion, suggesting a plausible mechanism through which exercise benefits IBD patients.

#### 2.4.3. Irritable Bowel Syndrome (IBS)

Irritable Bowel Syndrome (IBS) is one of the most diagnosed gastrointestinal disorders, being related to the manifestation of abdominal pain and discomfort and changes in bowel habits, and can be mixed, diarrheal, or constipation-related [113]. Patients with IBS also suffer from extra-intestinal symptoms such as migraine, depression, fatigue, and fibromyalgia [114].

Some lifestyle interventions, such as physical exercise, have been suggested to improve gastrointestinal and extra-intestinal symptoms [115]. The mechanisms by which training improves the symptoms of patients with IBS would be related to changes in intestinal blood flow, neuroendocrine and immunological changes, changes in intestinal motility, reduced stress, and improved well-being promoted by exercise [116].

Patients with IBS, when advised weekly to increase cardiorespiratory fitness by practising 20–60 min of moderate to vigorous physical activity 3 to 5 times a week, showed a significant improvement in these patients’ symptom scores after 12 weeks of counselling [117]. In another study, Johannesson et al. [118] also found improvement in gastrointestinal and psychological symptoms, such as fatigue, depression, and anxiety.

The attenuation of gastrointestinal symptoms in patients with IBS after aerobic training for 24 weeks may be due to the attenuation of the inflammatory process, with a reduction in plasma concentrations of cytokines (IL1β, IL-6, IL-8, IL-10 and TNF-α), reduction in oxidative stress markers, such as xanthine oxidase (XO), plasma malondialdehyde (MDA), and nitric oxide (NO), and increased antioxidant activity, such as superoxide dismutase (SOD), catalase (CAT), and glutathione peroxidase (GPx) [119,120].

Various training modalities (yoga, walking exercises, swimming, cycling, and other sports activities) have shown an effect. They can be considered an affordable and effective therapy for treating and managing IBS [119]. Notably, the literature on the subject has focused more on evaluating the effects of aerobic training on anaerobic training, with data on the latter still scarce. In its guidelines, the American College of Gastroenterology [121] hypothesizes that physical exercise can be an adjunctive therapy for IBS. However, evidence from clinical trials is still scarce, and it isn’t easy to establish a recommendation.

#### 2.4.4. Colorectal Cancer

Colorectal cancer is one of the most diagnosed cancers in both men and women in Brazil and worldwide, encompassing tumours that originate in the colon, rectum, and anus [122,123]. Its incidence rate is increasing considerably, with an estimated 2.5 million survivors expected by 2035 [124]. Among the risk factors associated with the manifestation of colorectal cancer are age over 50, poor lifestyle and dietary habits, economic development, obesity, physical inactivity, smoking, excessive alcohol consumption, diets high in red or processed meats, fats, sugary foods, and refined grains, and diets low in calcium, fruits, fibre, and vegetables [125].

Regular physical activity and exercise are also associated with a lower risk of colorectal cancer and other types of cancer through various mechanisms, such as the regulation of the cell cycle, improved immune function, protective action in the gastrointestinal tract via mediators secreted by skeletal muscle, mainly via myokines and exosomes, and increased antioxidant and anti-inflammatory responses [126]. Mechanistically, the main effects of colorectal cancer prevention linked to physical exercise include the redirection of insulin-like growth factor (IGF), reduced inflammation, cell death via apoptosis, epigenetic changes, and regulation of leptin and ghrelin levels [127].

Research has shown the benefits of physical exercise in treating patients with colorectal cancer, improving physical conditioning, functional and mental capacity, stress, and quality of life, enhancing immune function, oxidative stress, and prognosis [78,127,128]. A supervised 18-week exercise program for colorectal cancer patients undergoing chemotherapy reduced fatigue and proved to be safe and feasible [129].

Neoadjuvant exercise, as a prehabilitation strategy, is associated with lower morbidity in patients with colorectal cancer, considering that the primary treatment for this type of cancer is surgical. Improvement in physical fitness components is linked to better recovery and a lower recurrence rate in colorectal cancer [130,131]. It is important to note that some adverse effects of oncological treatments include peripheral neuropathy, fatigue, cardiovascular risk, pulmonary complications, immune and endocrine dysfunction, gastrointestinal motility alterations, anxiety, depression, and muscle weakness [132]. Thus, physical training can alleviate these symptoms and improve the quality of life in patients undergoing chemotherapy [127,133].

Among the mechanisms involved in the positive effects of physical exercise on colorectal cancer prognosis are modulation of the gut microbiota, with increased production of short-chain fatty acids that have anti-inflammatory effects, an increase in the population of Bifidobacterium, and a reduction in species such as *Fusobacterium nucleatum* and lipopolysaccharide-producing bacteria, which are pro-inflammatory, promoting increased intestinal permeability and metastasis [134].

Regarding the types of physical exercise and studies on other conditions that affect the gastrointestinal tract, most research has focused on the benefits and protocols of aerobic exercise. There is still a significant gap in clinical trials investigating the effects and establishing recommendations for resistance training in patients with colorectal cancer [127].

#### 2.4.5. Summary Recommendations of Physical Exercise in GI Dysfunctions

Based on the literature discussion, physical exercise can benefit various gastrointestinal diseases with appropriate intensity and modality.

- In patients with IBD, including Crohn’s disease and ulcerative colitis, walking or light-to-moderate running (60% of maximum heart rate) three times per week for 10 weeks improves the quality of life without exacerbating inflammation, while moderate-intensity aerobic and resistance training can help reduce inflammatory markers.
- For individuals with IBS, moderate aerobic activities such as walking, light running, and swimming aid in reducing abdominal pain and improving intestinal motility, whereas high-intensity exercise may worsen symptoms.
- In GERD, low-to-moderate-intensity exercises such as walking, light weight training, and yoga support weight management and diaphragm strengthening, while high-impact exercises and those performed right after meals should be avoided.
- In colorectal cancer, both prevention and post-treatment, regular aerobic exercise and moderate resistance training reduce inflammatory markers and the risk of recurrence.

Adjusting exercise intensity and frequency to maximize benefits without worsening gastrointestinal symptoms is essential in all these conditions.

## 3. Conclusions

In summary, the study of physical exercise on digestive physiology is quite old. However, this theme has been explored better in recent years, given the new methodologies for investigating the various intestinal segments. In addition, with techniques such as molecular biology and the study of specific gastric receptors and hormones, it was possible to understand better the mechanisms involved in gastrointestinal changes associated with physical exercise at various intensities, both in experimental models and clinical studies in humans.

## Figures and Tables

**Table 1:** Summary of the main benefits and risks of exercise on the gastrointestinal tract. Adapted from Silva et al. [9].

	Benefits	Risks
**Esophagus**	None	Acid reflux induction
**Stomach**	Light Exercise Accelerates Gastric Emptying	High-Intensity Exercise Delays Gastric Emptying and Inhibits Acid Production
**Small bowel**	None	High-Intensity Exercise Interferes with Absorption and Induces Bleeding
**Colon**	Exercise Reduces the Risk of Colon Cancer and Diverticulitis	High-Intensity Exercise Induces Bleeding
**Liver**	None	None

**Table 2:** Physical exercise perceptions on gastrointestinal disorders.

Category	Effects Observed	Evidence	Conclusions
Gastroesophageal reflux	Symptoms of gastroesophageal reflux are commonly observed during exercise.	Suggestive	Some activities like running can cause transient lower esophageal sphincter relaxation, increased abdominal pressure, and decreased esophageal clearance during exercise, leading to reflux episodes without pathological evidence [54].
	Exercise can exhibit protection against GERD.	Convincing	In two systematic reviews, higher levels of recreational physical activity reduce the risk of GERD. One also found a reduced risk of esophageal adenocarcinoma [55,56].
Gastrointestinal motility	Exercise alters gastric emptying and digestion.	Unclear	The effects seem to be different according to exercise intensity. Light exercise seems related to gastric emptying acceleration, while high-intensity exercise delays. Acute and chronic exercise also have been implicated in different effects on gastric emptying. Ingested food/drink or supplements can influence gastric emptying responses to exercise. Evidence also focuses on the capacity of exercise to regulate gastric emptying in some clinical conditions, such as chemotherapy, IBD, diabetes mellitus, and hypertension [48,53,57,58,59,60,61].
	Exercise changes intestinal motility.	Unclear	Some evidence shows that exercise can alter gastrointestinal motility by changing the myoelectric activity of gastrointestinal cells, reducing motility, while other evidence found no alterations [44,62,63]. These effects can also be mediated by diet before the training and mechanical effects of exercise.
Constipation	Exercise can relieve chronic constipation.	Limited evidence	In a systematic review and meta-analysis, the authors found that exercise significantly improved the symptoms of patients with constipation, and aerobic exercise positively impacted constipation. However, the methodological issues and standardization of exercise protocols can limit scientific evidence [64].
Cancer risk	Exercise may reduce digestive cancer risk.	Convincing	Systematic reviews and metanalysis found that exercise protects against digestive cancers [65,66].
Gastrointestinal bleeding	Strenuous exercise can cause bleeding in the gastrointestinal tract	Suggestive	Athletes can face some GI bleeding from the upper or lower GI tract, with severity linked to effort intensity and duration. Key factors include splanchnic hypoperfusion, GI wall trauma, and NSAID use. Proper nutrition, hydration, exercise regulation, and supplements can alleviate symptoms such as nausea, vomiting, cramping, diarrhea, and potential hemorrhage [67].

Legend: GERD: gastroesophageal reflux disease; GI: gastrointestinal tract; IBD: inflammatory bowel disease; NSAIDs: non-steroidal anti-inflammatory drugs.

**Table 3:** Results of clinical trials that evaluated the therapeutic effect of physical exercise on gastrointestinal diseases.

Author/Year	Localization	Exercise Group	Control	Type of Disease	Exercise Protocol	Duration	Main Results
**Brown et al. (2018)** [78]	USA	Low dose (n = 14) High dose (n = 13)	13	Patients with colon cancer in stages I-III	Treadmill aerobic exercise (50–70% HRmax)	Low-dose group (150 min/week) High-dose group (300 min/week) 6 months of intervention	A questionnaire measured the best score on the quality-of-life test. There was no effect on gastrointestinal symptoms.
**Collings et al. (2003)** [79]	USA	Athletes Race (n = 10) Cycling (n = 10) Weightlifting (n = 10)	-	GERD	Aerobic exercise 65% (60 min) and 85% (20 min) of HRmax	Fasting exercises and postprandial (45 min after meals) 60 min Rest 20 min	Weightlifters—more heartburn and reflux. Runners—mild symptoms and moderate reflux. Cyclists—mild symptoms and reflux. Strenuous exercise induces significant reflux and related symptoms in conditioned people.
**Cronin et al. (2019)** [80]	Ireland	13	7	Crohn’s Disease and Ulcerative Colitis	A combined program of aerobic and resistance training of moderate and progressive intensity	8 weeks of intervention	Improved aerobic capacity. Improved body composition. There was no difference in disease activity. There were no changes in the α and β-diversities of the intestinal microbiota.
**Daley et al. (2008)** [81]	England	28	28	Patients with IBS	Customized training	12 weeks	There was no difference in the quality-of-life score between the groups. The trained group showed significant improvement in the symptoms of constipation.
**Klare et al. (2015)** [82]	Germany	15	15	Crohn’s Disease and Ulcerative Colitis	Moderate intensity running	10 weeks	Improved quality of life. There was no exacerbation of gastrointestinal symptoms.
**Mendes-Filho et al. (2014)** [83]	Netherlands	10 healthy volunteers	-	Investigation of possible effects on GERD	After a standard meal and rest Aerobic exercise (treadmill) 60% (30 min) and 85% (20 min) of HRmax	30 min Rest 20 min	Longer time with esophageal pH <4, frequency, and duration of reflux episodes. Decreased contractility and duration of peristaltic contractions. A hiatal hernia was detected during the exercise but not during rest.

Legend: GERD: gastroesophageal reflux disease; IBS: irritable bowel syndrome.
